# Identification of Circular RNAs in Kiwifruit and Their Species-Specific Response to Bacterial Canker Pathogen Invasion

**DOI:** 10.3389/fpls.2017.00413

**Published:** 2017-03-27

**Authors:** Zupeng Wang, Yifei Liu, Dawei Li, Li Li, Qiong Zhang, Shuaibin Wang, Hongwen Huang

**Affiliations:** ^1^Key Laboratory of Plant Resources Conservation and Sustainable Utilization, South China Botanical Garden, Chinese Academy of SciencesGuangzhou, China; ^2^Guangdong Provincial Key Laboratory of Applied BotanyGuangzhou, China; ^3^College of Life Sciences, University of Chinese Academy of SciencesBeijing, China; ^4^Key Laboratory of Plant Germplasm Enhancement and Specially Agriculture, The Chinese Academy of SciencesWuhan, China

**Keywords:** kiwifruit, circRNAs, PSA, plant defense, WGCNA

## Abstract

Research studies have recently focused on circle RNAs (circRNAs) in relation to their regulatory functions in animals. However, the systematic identification of circRNAs in plants, especially non-model plants, is limited. In addition, raw report on the prediction of the potential role of circRNAs in plant response to pathogen invasion is currently available. We conducted the systematic identification of circRNAs from four materials originating from three species belonging to genus *Actinidia* under different situations using ribosomal RNA (rRNA) depleted RNA-Seq data. A total of 3,582 circRNAs were identified in *Actinidia*, of which 64.01, 21.44, and 14.55% were intergenic circRNAs, exonic circRNAs, and intronic circRNAs, respectively. Tissue-specific expression of circRNAs was observed in kiwifruit, and a species-specific response was detected when infected with *Pseudomonas syringae* pv. *actinidiae* (Psa), which is the causative agent of kiwifruit bacterial canker disease. Furthermore, we found that both exonic and intronic circRNAs were significantly positively correlated to parent protein-coding genes, and intronic circRNAs are a class of highly remarkable regulators the parent genes comparing to that of exonic circRNAs. Expression and weighted gene co-expression network analysis (WGCNA) identified a set of circRNAs that were closely associated with plant defense response. The findings of the presents study suggest that circRNAs exhibit tissue- and species-specific expression, as well as play an important role in plant immune response.

## Introduction

A diverse class of non-coding RNAs (ncRNAs) exists in eukaryotic cells, and a large proportion of known ncRNAs are known to undertake important biological functions (Morris and Mattick, 2014). Amongst ncRNAs, circular RNAs (circRNAs), which were initially reported almost four decades ago (Hsu and Coca-Prados, 1979; Arnberg et al., 1980) and have often been regarded as products of *mis*-splicing events (Cocquerelle et al., 1993), have received increasing attention in recent years partly due to progress in high-throughput sequencing techniques and high-efficiency bioinformatics approaches (Memczak et al., 2013; Guo et al., 2014; Fan et al., 2015; Lu et al., 2015; Ye et al., 2015). CircRNAs exist in both unicellular and multicellular organisms, and their abundance and evolutionary conservation among various species are suggestive of their important yet undiscovered functions (Hansen et al., 2013; Memczak et al., 2013; Lu et al., 2015).

CircRNAs are produced from precursor mRNAs (pre-mRNAs) through backsplicing (also called head-to-tail splicing) in which an upstream 3′ splicing acceptor site is joined to a downstream 5′ splicing donor site (Ye et al., 2015). Alternative mechanisms for generating circRNAs from both introns and intergenic regions have also been reported (Zhang et al., 2013; Jeck and Sharpless, 2014; Lasda and Parker, 2014). It has been shown that both canonical splice signals and canonical spliceosomal machinery are needed for effective backsplicing (Chen and Yang, 2015). These result in competition between canonical splicing and backsplicing in cells, which can explain the generally lower abundance of circRNAs compared to their linear mRNA counterparts when spliceosomes are unfavorably assembled at backsplicing sites (Chen, 2016). Both *cis*-regulatory elements and *trans*-acting factors are essential in the control of splicing, which in turn promotes circRNA biogenesis (Chen, 2016). A recent study has suggested that alternative splicing (AS) events are also involved into the biosynthesis of circRNAs (Chen and Yang, 2015), thus further increasing the complexity of circRNA transcriptions.

The general functions of most circRNAs remain far from clear, although some circRNAs have been shown to play important regulatory roles in gene expression (Hansen et al., 2013; Memczak et al., 2013; Conn et al., 2015; Kashi et al., 2015; Venø et al., 2015). A circRNA, ciRS-7, which is highly expressed in both human and mice, has been found to act as an efficient microRNA sponge for miR-7 (Hansen et al., 2013). Similarly, a class of circRNAs that are circularized with introns and retained between exons in human cells enhances the expression of their parental genes in *cis* via specific RNA-RNA interactions (Li et al., 2015). CircRNAs could also affect AS, leading to altered gene expression in humans because their formation is positively correlated with exon skipping in linear mRNAs (Chen, 2016). In plants, the identification and functional characterization of circRNAs remain extremely rare, despite recent work involving model plants such as rice and *Arabidopsis* that demonstrated that circRNAs are widely distributed and their features are apparently distinct from those in animals (Lu et al., 2015; Ye et al., 2015). Moreover, the expression of circRNAs in both animals and plants are often described in spatial/temporal specific patterns, and circRNAs responsible for alternative biological processes are possibly highly specific (Guo et al., 2014; Conn et al., 2015; Fan et al., 2015; Ye et al., 2015; Sablok et al., 2016).

Kiwifruit (*Actinidia chinensis* Planchon) is an important specialty fruit crop and is currently grown commercially worldwide. However, the recent outbreak of kiwifruit canker disease caused by *Pseudomonas syringae* pv. *actinidiae* (Psa) have severely affected global kiwifruit industry (Spinelli et al., 2011). A number of strategies for canker disease control have been presented (Reglinski et al., 2013); however, none have been shown to be effective in inhibiting Psa from invading into kiwifruit host tissues and cells, and this may be partly due to our limited understanding of its host-pathogen molecular interactions. Based on phylogenetic analysis, Psa strains can be grouped into five biovars (Biovars 1–5; Fujikawa and Sawada, 2016), in which biovar 3 has the high virulence and is the causative agent of the current outbreaks of kiwifruit canker disease. Previous research investigations have revealed that plant recognition receptors proteins (PRRs) genes, resistance (R) genes, and transcriptional factors (TFs) significantly affect plant immune responses to pathogen invasion (Chisholm et al., 2006; Pandey and Somssich, 2009; Kazan and Lyons, 2014). However, the relationship between circRNAs and immune-related genes is unclear, and the potential role of circRNAs in plant immune response remains elusive.

Here, we identified circRNAs in kiwifruit plants by using the ribosomal RNA (rRNA) depleted RNA-Sequencing (RibominusSeq) technique (Memczak et al., 2013; Lu et al., 2015; Ye et al., 2015). We determined that the circRNAs in kiwifruit are highly expressed in a tissue-specific pattern. With inoculation of a high virulent Psa strain on leaves of three different *Actinidia* taxa, we further recognized the species and Psa invading-stage specific expression of circRNAs. By using weighted gene co-expression network analysis (WGCNA; Langfelder and Horvath, 2008) to infer possible functions of circRNAs, we finally identified a set of circRNAs that are potentially associated with kiwifruit responses to Psa infections.

## Materials and methods

### Ribominusseq library construction and sequencing

To investigate the expression pattern of circRNAs in different kiwifruit tissues, we constructed RNA libraries from leaf, root, and stem tissues from tissue culture seedlings of the *A. chinensis* (Ac)-derived cultivar “Hongyang” (AH; referred here as tissue-dataset, Supplementary Table 1). Two biological replicates of each tissue were prepared.

To study the specific expression of circRNAs in relation to different *Actinidia* taxa that cause Psa infection, we constructed RNA libraries from the leaves of tissue culture seedlings of three species: two Ac-derived cultivars [the AH abovementioned and a cultivar “Jinyan” (AJ)], the species *A. eriantha* (Ae), and *A. arguta* (Aa; taxon-dataset, Supplementary Table 1). We used a highly virulent Psa strain, C48, which was originally isolated from a kiwifruit orchard in the Anhui Province, China, in inducing typical canker disease on the leaves of the study materials. The strain belongs to the biovar 3 clade of the whole Psa phylogeny, which has high virulence that is responsible for the current outbreak of bacterial canker disease in kiwifruit (McCann et al., 2013). Incubation experiments were performed using these plant materials in a plant growth chamber under controlled temperature (day: 25°C, night: 20°C) and humidity (70–100%). A bacterial suspension, containing ~108 cells/mL Psa, was prepared from overnight culture and used for the inoculation. The bacterial suspension was injected into the petioles of three leaves from a single plantlet using sterilized syringes. Leaf tissues of the four materials from the following three stages were collected for library construction: day 0 post incubation (DPI; thus Psa-free), as well as 2 and 14 DPIs (reflecting the initial and top infection of Psa on kiwifruit leaves, respectively). Two biological replicates of each sample at each sampling stage were harvested for library construction.

To directly trace and observe Psa in kiwifruit leaves, we labeled the Psa strain C48 with GFPuv by transforming a stable and broad-host-range plasmid vector (pDSK-GFPuv) using Bio-Rad MicroPulser (Bio-Rad, USA). We calculated the leaf area based on the green fluorescence emitted by each sample using ImageJ (http://imagej.net).

Total RNA of each sample was isolated using HiPure Plant RNA Mini Kit (Magen, Guangzhou, China) according to the manufacturer's instructions. All total RNA samples were treated with the RQ1 DNase (Promega, USA) to remove any contaminating DNA. A total of 30 RNA libraries were constructed using NEBNext® Ultra™ Directional RNA Library Prep Kit for Illumina® (NEB, USA) following manufacturer's recommendations, and index codes were added to ascribe sequences to each sample.

The resulting libraries were initially sequenced on an Illumina Hiseq 2000 instrument (Illumina, USA) that generated paired-end reads of 125 base pairs (bp) in length. The raw sequencing data were submitted to the National Center for Biotechnology Information (NCBI) Sequence Read Archive with a Bioproject ID PRJNA328414, and sample accession IDs of SRS1552843, SRS1552846-SRS1552860, SRS1552862-SRS1552865, and SRS1552867-SRS1552876.

### Identification of circular RNAs

We filtered the raw reads in fastq format to remove reads containing adapter or ploy-N and low quality reads using in-house Perl scripts. For genome-wide identification of circRNAs, we first mapped RibominusSeq reads to a combination of kiwifruit genome references derived from Ac (Huang et al., 2013) and the Psa strain NZ13, respectively (McCann et al., 2013), using BWA-MEM (v0.7.13; Li, 2013) with the parameter *T* = 19. The SAM file of alignment was then inspected by using CIRI (v1.2; Gao et al., 2015) to identify circRNA candidates. Briefly, CIRI scans the SAM alignment twice, in which the first scan detects junction reads with paired chiastic clipping (PCC) signals that reflect a circRNA candidate, and the second scan detects additional junction reads and performs further filtering to eliminate false-positive candidates that result from incorrectly mapped reads of homologous genes or repetitive sequences (Gao et al., 2015). We further manually filtered out circRNAs by only retaining those that were detected in both biological replicates. The final set of circRNAs was divided into three groups, namely, exonic circRNAs, intronic circRNAs, and intergenic circRNAs on the basis of their genomic region origin.

To identify and annotate protein-coding transcripts from our transcriptome, we used STAR (Dobin et al., 2013) to align reads to the kiwifruit reference genome and then assemble transcripts using StringTie (Pertea et al., 2015). We used Annocript (Musacchia et al., 2015) to annotate protein-coding transcripts and then used cuffcompare (Trapnell et al., 2012) to optimize the annotations based on the reference.

### Validation of circular RNAs

To validate the identified circRNAs in kiwifruit, we extracted total RNA from kiwifruit seedlings grown under the same conditions as those used for RibominusSeq sequencing. Genomic DNA of kiwifruit leaves was also isolated using HiPure Plant DNA Mini Kit (Magen, Guangzhou, China). The first-strand cDNA was synthesized from 1 μg of total RNA with random primers using the TransScript One-Step gDNA Removal and cDNA Synthesis SuperMix (TRANSGEN BIOTECH, Beijing, China). A total of 80 circRNAs, including 20 exonic circRNAs, 20 intronic circRNAs, and 40 intergenic circRNAs were randomly selected for validation of polymerase chain reactions (PCRs) and Sanger sequencing. A set of divergent primers, which are also called outward-facing primers (Supplementary Table 3), were designed using the Primer3Plus software online (http://www.bioinformatics.nl/cgi-bin/primer3plus/primer3plus.cgi) and further synthesized commercially (Sangon Biotech Co., Ltd., Shanghai, China). Both cDNA and gDNA were used as PCR templates for each divergent primer pair. All PCR products were further separated in a 1% agarose gel for subsequent purification and Sanger sequencing commercially. To verify the expression of circRNAs, we carried out quantitative PCR (qPCR). The qPCR was carried out in a total volume of 20 μL, containing 10 μL of Tip Green qPCR SuperMix (TransGen Biotech Co., Ltd.), 0.2 μM of each primer, 1 μL of 1:5 diluted cDNA and 8.2 μL ddH_2_O. Thermal cycling consisted of a hold at 94°C for 30 s, followed by 40 cycles of 94°C for 5 s, and 60°C for 30 s. The temperature was then gradually raised, by 0.5°C every 10 s, to perform melting-curve analysis. Each sample was amplified in triplicate, and all PCR reactions were performed on the LightCycler 480 (Roche, Basel, Switzerland). The ΔΔCt method was employed with kiwifruit actin (Achn107181) as endogenous control genes.

### Expression analysis of circRNAs

To compare the expression of circRNAs across various kiwifruit tissues and taxa, we calculated the accounts of backspliced reads from the CIRI results for each circRNA that was normalized by using the total sequencing reads in a corresponding sample data set (defined as reads per million mapped reads, RPM) as an indicator of their expression levels (Song et al., 2016). To calculate the expression of protein-coding transcripts, read numbers of each transcript were retrieved using RSEM (v1.2.17; Li and Dewey, 2011), and then used as input to calculate the expected number of fragments per kilobase of transcript sequence per million base pairs sequenced (FPKM). To identify differentially expressed protein-coding transcripts, analysis of pairwise differential expression was performed using DESeq2 (Love et al., 2014) on the basis of the count matrices. We classified transcripts as differentially expressed when the adjusted *p*-value was < 0.05 (FDR < 5%) and the moderate fold change was >1. We further performed principal component analysis (PCA) for all samples within the tissue-dataset and the taxon-dataset, respectively based on the RPM matrix using the package “pca3d” in R (Weiner, 2015). The differentially expressed circRNAs during disparate stages on the basis of RPM matrix were identified using the paired *t*-test with a *p* < 0.05 for samples in the taxon-dataset. Sample clustering were performed using TM4 (v4.9; Saeed et al., 2003) on the basis of circRNA expression matrix.

To investigate expression correlation of exonic/intronic circRNAs and corresponding parent protein-coding genes from which exonic/intronic circRNAs were derived, we first retrieved the expression matrix of circRNA-parent gene pairs using in-house Perl scripts. The pairwise expression correlation between circRNAs and their corresponding parent genes was computed using Spearman's method and marked as *r*_s_. Protein-coding parent genes that were significantly correlated to corresponding circRNAs (*r*_s_ > 0.5 or *r*_s_ < −0.5) were selected for further Gene Ontology (GO) enrichment analysis using “clusterProfiler” package (Yu et al., 2012) in R. Significant enriched terms were identified by *q* < 0.05 (Yu et al., 2012).

### Co-expression of protein-coding genes and circRNAs

We used WGCNA (Langfelder and Horvath, 2008) to assess the potential function of circRNAs that were involved in Psa infections in kiwifruit plants. We first combined the expression matrix of both protein-coding genes and circRNAs (including 584 circRNAs and 8,700 protein-coding transcripts) as the input file for WGCNA analysis to identify modules of genes with strong co-expression. Next, we calculated a series soft thresholding power (from 1 to 20) following scale-free topology criteria, and we here employed a soft power value of 9 to identity modules. The trait profile, which included the leaf area with green fluorescence signals that reflected Psa infection (trait 1), sampling stages (trait 2). and taxa examined (trait 3), was used as a respective input files for the detection of significant relationships (*p* < 0.05) among traits and the eigengene of each module. The eigengene was defined as the first principal component of a given module and considered a representative of the gene expression profiles in a module (Langfelder and Horvath, 2008). We retained transcripts of R genes, PRR genes, and TFs within each module based on gene annotations. Protein-coding genes in each module were retrieved for GO enrichment analysis, and the functions of circRNAs were thus inferred. The network result of each WGCNA module was visualized using Cytoscape (v2.8.3; Demchak et al., 2014).

## Results

### CircRNAs identified in kiwifruit

We performed inoculation experiment to investigate the kiwifruit response to the invasion of Psa. Our results showed divergent symptoms on leaves of different *Actinidia* species at both 2 and 14 DPI with the Psa infection (Supplementary Figure 1). We found typical leaf symptoms (leaf damage and shrinkage with the presence of necrotic lesions) were present in Ac (including cultivar AH and AJ) at 14 DPI, which were similar to those observed in Psa-infected orchards. In contrast no obvious symptoms were appeared in Ae and Aa at the same stage (Supplementary Figure 1). Additionally strong green fluorescence was recorded in Ac at 14 DPI and the green fluorescence was weak on leaves of Ae and Aa although it was detectable (Supplementary Figure 1).

To obtain sufficient RNA-Seq reads to increase the likelihood of detecting circRNAs, we deep-sequenced each rRNA-depleted library with an average data volume of about 12 Gb, thereby yielding a total of 2,884 million paired-end reads with a size of 125 bp. Using the circRNA identification tool CIRI and further manual filtering to exclude false-positive candidates, we identified a total of 3,582 circRNAs from all 30 libraries, including 1,230 circRNAs from the tissue-dataset that was derived from six libraries constructed from different kiwifruit tissues, and 2,914 circRNAs from the taxon-dataset with 24 Psa-infected leaf libraries from a combination of four materials (from three species) and three sampling stages (Supplementary Table 1).

**Figure 1:**
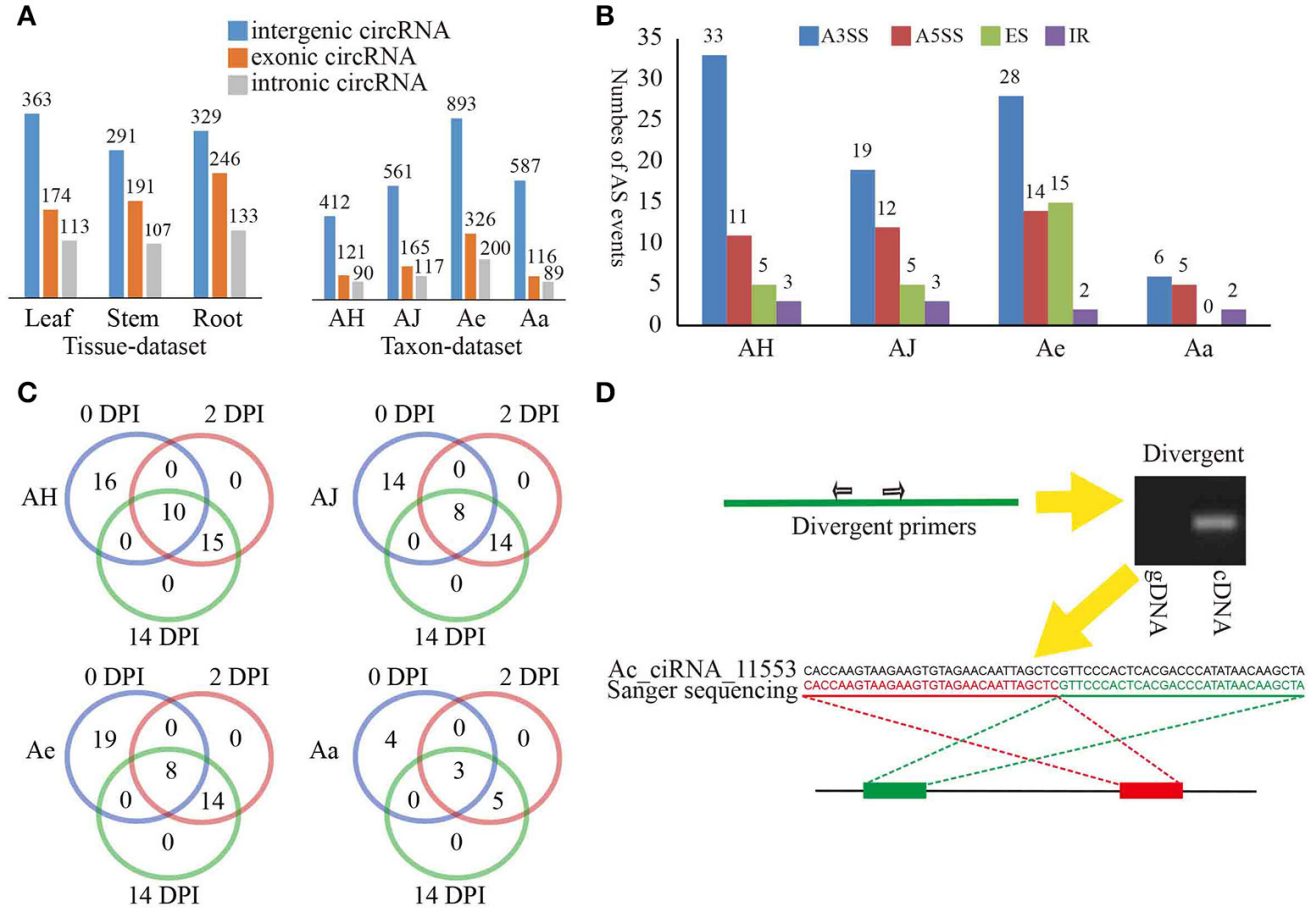
**Overview of kiwifruit circRNAs and circRNA validation. (A)** Distribution of different type circRNAs classified in various tissues and materials. **(B)** Distribution of different type AS events of circRNAs in various materials. A3SS, alternative 3′ splice site; A5SS, alternative 5′ splice site; ER, exon skipping; IR, intron retention. **(C)** Distribution of different type AS events of circRNAs at various sampling stages for four *Actinidia* species/cultivars. **(D)** An example of circRNA validation using PCR and Sanger sequencing. Red and green rectangles represent downstream and upstream sequences in the genome respectively. Upper left panel, a model showing divergent primers for amplification of circRNA. Upper right panel, an example showing that divergent primers amplified circRNA in cDNA but not in genomic DNA; Lower panel, a Sanger sequencing example an *Actinidia* circRNA Ac_ciRNA_11553.

We classified the identified circRNAs into three groups, namely, exonic circRNAs, intergenic circRNAs, and intronic circRNAs. Interestingly, in both the tissue- and taxon-data sets, intergenic circRNAs predominated (51 and 67%, respectively) compared to the exonic and intronic circRNAs (Figure 1A). These results were not identified with earlier findings that circRNAs in plants are mainly derived from coding regions of both monocot (*Oryza sativa*) and dicot (*Arabidopsis thaliana*) plants (Ye et al., 2015). Additionally, we also found that the same genomic locus can produce multiple circRNAs based on AS. We identified a total of 163 AS events, in which an alternative 3′ splice site (A3SS) event was the most prevalent (63.46, 48.72, 47.46, and 46.15% in AH, AJ, Ae, and Aa, respectively; Figure 1B). For the four materials examined in the taxon-dataset, the majority of AS events (56, 69, and 77% in AJ, Ae, and Aa, respectively) occurred in circRNAs that originated from protein-coding genes (including exonic and intronic circRNAs) except for that in AH (Supplementary Figure 2A).

During Psa invasion, the AS events occurring across different stages were essentially stable, particularly for the 2 and 14 DPI time points (Supplementary Figure 2B). In addition, these AS events at both 2 and 14 DPI were the same in all four materials investigated (Figure 1C), thereby suggesting potential stable expression of circRNAs during Psa infection. We further found that those protein-coding genes that constantly underwent AS at both DPI were closely related to plant defense responses (Supplementary Table 2). For example, Ac_ciRNA_04842, which was spliced from the *Achn372061* gene in Ae, and *Achn372061* were derived from a calcium-dependent protein kinase 4 (CDPK4) gene. Previous studies have verified that calcium-dependent protein kinases (CDPKs) play an essential role in plant defensive response (Romeis et al., 2001). Additionally, the expression of *Achn372061* was significantly and positively correlated to the expression of Ac_ciRNA_04842 (expression correlation: 0.963), illustrating that circRNA can enhance plant resistance to pathogen via regulation of expression of related protein-coding genes (Supplementary Table 2).

To confirm the validity of circRNAs identified from the RNA-Seq data, we randomly selected a subset of 80 circRNAs for experimental validation using reverse transcription (RT)-PCR. A pair of divergent primers (Supplementary Table 3) was designed for each circRNA, and both cDNA and gDNA were used as template for PCR amplification (Figure 1D). The expected results would be positive and negative amplification in cDNA and gDNA, respectively. Approximately 68 of the 80 circRNAs were confirmed (Supplementary Table 3, see an example in Figure 1D). The validation rates of different types of circRNAs were similar, with 80% for exonic circRNAs, 75% for intronic circRNAs, and 87.5% for intergenic circRNAs, thereby suggesting stable expression of different types of circRNAs in kiwifruit tissues and taxa. All PCR products were further validated by Sanger sequencing. For example, Ac_ciRNA_11553 is an intergenic circRNA, and the junction site was confirmed by Sanger sequencing and RNA-Seq (Figure 1D).

### Diverse expression patterns of circular RNAs in kiwifruit

**Figure 2:**
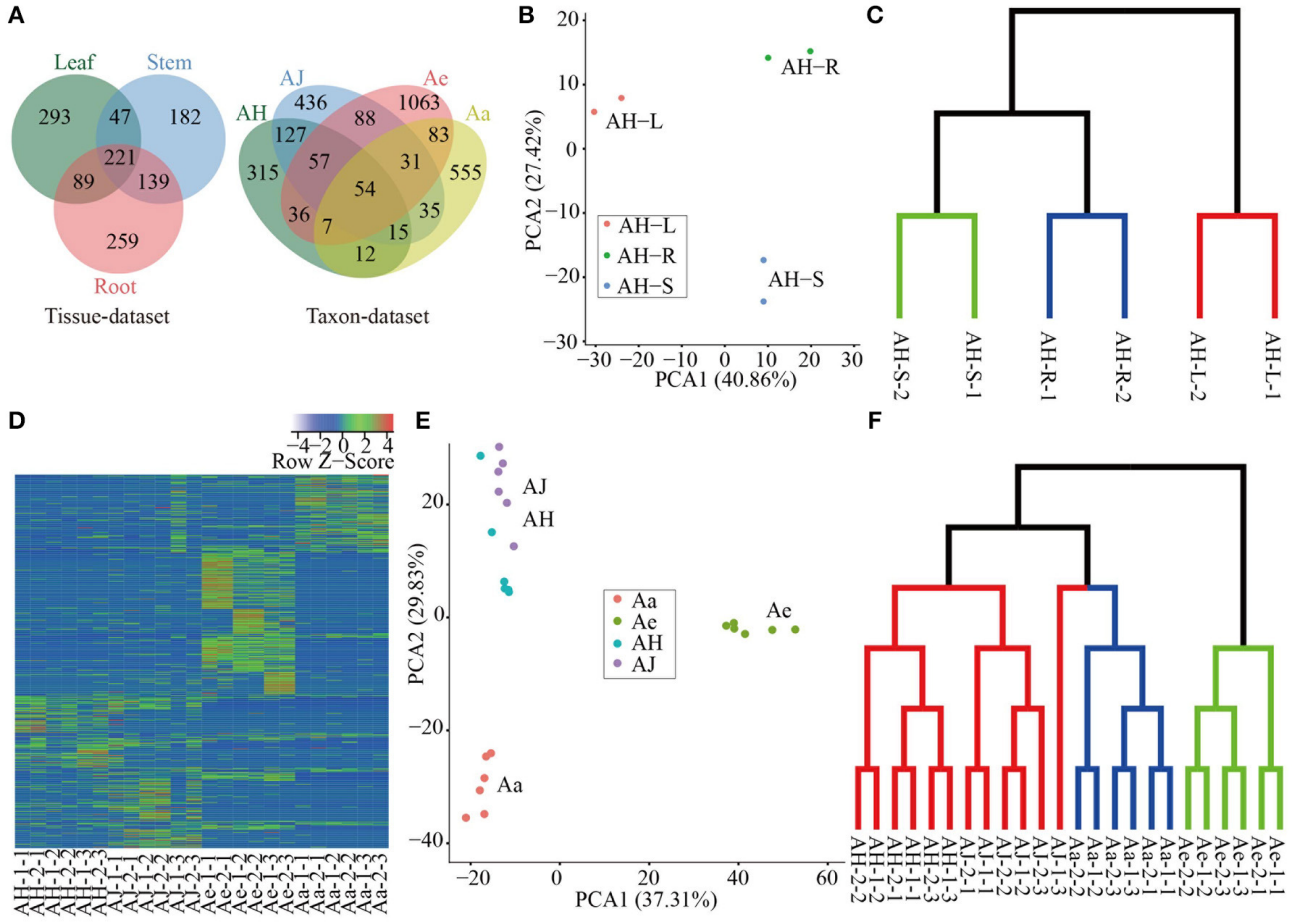
**Expression analysis of circRNAs in different tissues and species. (A)** Venn diagram of circRNA distribution in a tissue dataset and taxon dataset respectively. **(B)** PCA of all samples based of RPM matrix in tissue-dataset, each dot represents one sample. Principal components one and two (PC1 and PC2) collectively explained 68.28% of the variance. **(C)** Sample clustering of different tissues based on RPM matrix of circRNAs. The green, blue and red clades represent samples of stem, root and leaf respectively. **(D)** Heatmap of expression of all circRNAs in all samples, each row and column represent one circRNA and one sample, respectively, the color represents Z-score transformed from RPM of circRNAs. **(E)** PCA of all samples based of RPM matrix in taxon-dataset, each dot represents one sample. The PC1 and PC2 collectively explained 67.14% of the variance. **(F)** Sample clustering of different materials at various sampling stages during Psa invasion. The green, blue, and red clades represent samples of Ae, Aa, and Ac (including AH and AJ) respectively.

We quantitated the expression of circRNAs in all samples and we found that all circRNAs were expressed (RPM > 0) in at least one sample (including two replicates, Supplementary Table 4). The expression of the majority of circRNAs was tissue- or taxon-specific (Figure 2A). In the tissue-dataset, we found that 59.67% of the circRNAs was expressed in only one tissue, whereas those presented across three tissues commonly only accounted for 17.97% of the total number of expressed circRNAs. On the basis of the RPM expression matrix (Supplementary Table 4), we performed PCA analysis of all circRNAs identified in the tissue-dataset, which resulted in three clear clusters representing each of the tissue samples examined and (Figure 2B). Further clustering using TM4 (v4.9; Saeed et al., 2003) revealed a similar pattern of strong tissue-specific expression (Figure 2C).

For circRNAs identified in the taxon-dataset with libraries constructed from Psa-infected leaves from different *Actinidia* species/cultivars, 81.3% of the circRNAs are specific to each of the four species/cultivars investigated (Figure 2A). Furthermore, for the three stages (0, 2, and 14 DPI) examined during Psa invasions on kiwifruit leaves, 58.59% (365), 68.33% (574), 49.75% (706), and 49.12% (389) circRNAs in AH, AJ, Ae, and Aa, respectively, were expressed in only one stage (Supplementary Figure 3), thereby suggesting that the expression pattern of circRNAs across different kiwifruit materials was also stage-specific. We identified a total of 584 differentially expressed circRNAs (Supplementary Table 5) during Psa infection, in which both the number and expression levels were highly heterogeneous, thereby increasing the diversity in the expression profiles of kiwifruit plants during Psa infection (Supplementary Figure 3 and Figure 2D). On the basis of the RPM expression matrix for circRNAs in the taxon-dataset, both PCA and clustering analysis identified three distinct groups that mainly reflected species differentiation (Figures 2E,F). Moreover, within each species, the samples were more easily grouped based on the stage examined (Figure 2F).

### Correlation of gene expression between circRNAs and parent genes

**Figure 3:**
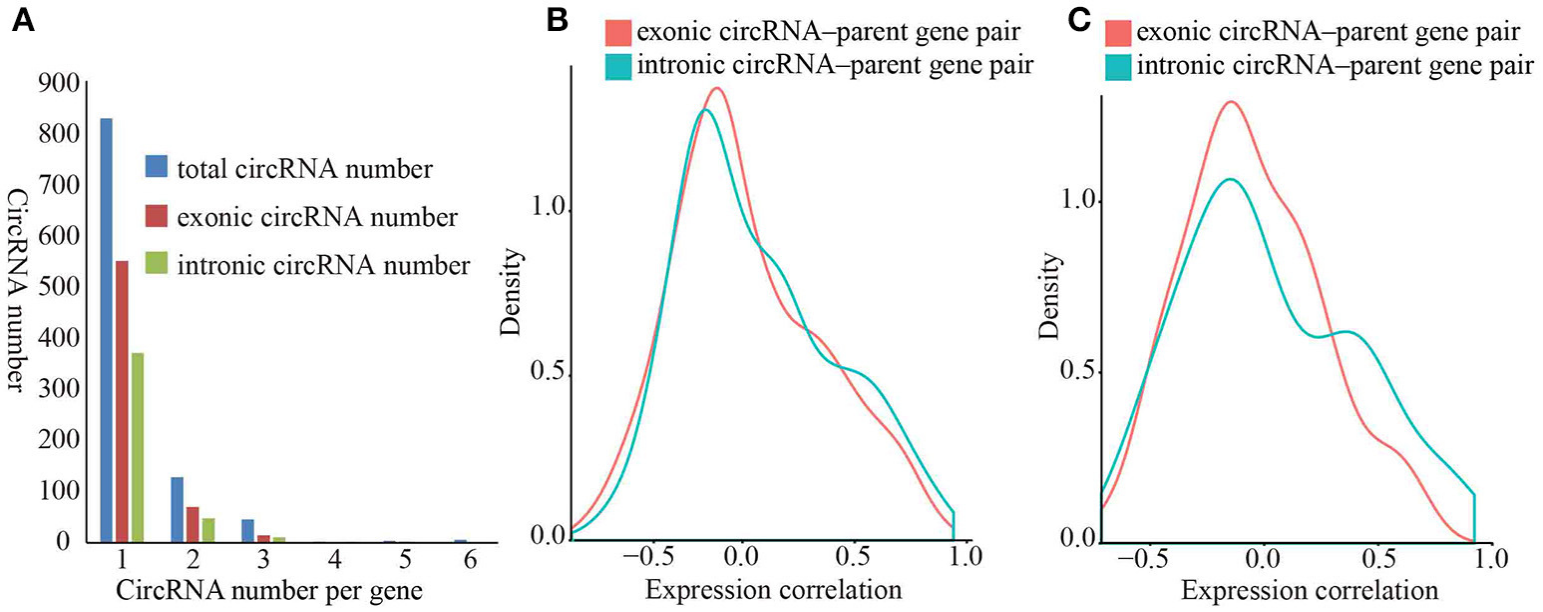
**Density diagram of expression correlation between circRNAs and parent genes. (A)** Distribution of protein-coding genes that originated from circRNAs. The blue, red and green bars represent total circRNA number, exonic circRNA number and intronic circRNA number respectively. **(B)** Density diagram of correlation between circRNAs and parent genes. The red and green lines represent correlation of exonic circRNA–parent gene pairs and intronic circRNA–parent gene pairs respectively. **(C)** Density diagram of correlation between circRNAs and protein-coding genes that produce both exonic circRNAs and intronic circRNAs. The red and green lines represent correlation of exonic circRNA–parent gene pairs and intronic circRNA–parent gene pairs respectively.

Exon-intron circRNAs can upregulate the expression of their parent protein-coding genes by interacting with U1 snRNP (Li et al., 2015). Both exonic and intronic region of protein-coding genes can form circRNAs. However, it is unclear whether circRNAs that originated from various sites of protein-coding genes impart different effects on the expression of their parent genes. To investigate whether exonic and intronic circRNAs have disparate effects on the expression of their parent genes, we first calculated pairwise expression correlations between exonic/intronic circRNAs and their parent genes in all samples. The expressions of both exonic and intronic circRNAs was more positively relevant to the expression of parent genes (Figure 3B), in which 15.33% of the exonic circRNAs-parent gene pairs and 19.64% of intronic circRNA-parent gene pairs showed a positive correlation coefficient (*r*_s_) with a *p* < 0.05 vs. 8.33 and 5.19% with a negative *r*_s_ (Supplementary Table 6). These results suggested that both exonic and intronic circRNAs could enhance the expression of their parent genes.

We observed a predominant tendency for positive correlation in the intronic circRNA-parent gene pairs than that in the exonic circRNA-parent gene pairs (Supplementary Table 6), thereby suggesting that intronic circRNAs remarkably affect the upregulating of their parent gene. To directly compare the effects of exonic and intronic circRNAs, we selected protein-coding genes that can simultaneously produce exonic and intronic circRNAs and then calculated the expression correlations of these gene pairs. We similarly found a predominant positive and negative expression correlation for intronic circRNA-parent gene pairs than that for exonic circRNAs-parent gene pairs (Figure 3C), in which 12.77 and 6.38% of intronic circRNAs-parent gene pairs had a positive/negative correlation coefficient, respectively, with a *p* < 0.05 vs. 5.15 and 5.15% of exonic circRNAs-parent gene pairs, respectively. These results suggested that intronic circRNAs play a more important role in expression regulation of parent genes in kiwifruit. Furthermore, we performed GO enrichment analysis of the corresponding parental genes that were significantly correlated to circRNAs (| *r*_s_ | > 0.5). We found that the GO terms of these parental genes were associated with various biological processes (Supplementary Table 7), thus suggesting that circRNAs potentially have diverse biological functions and no preferential origin for circRNA was established in relation to any parental gene function.

### Co-expression of protein-coding genes and circRNA genes

**Figure 4:**
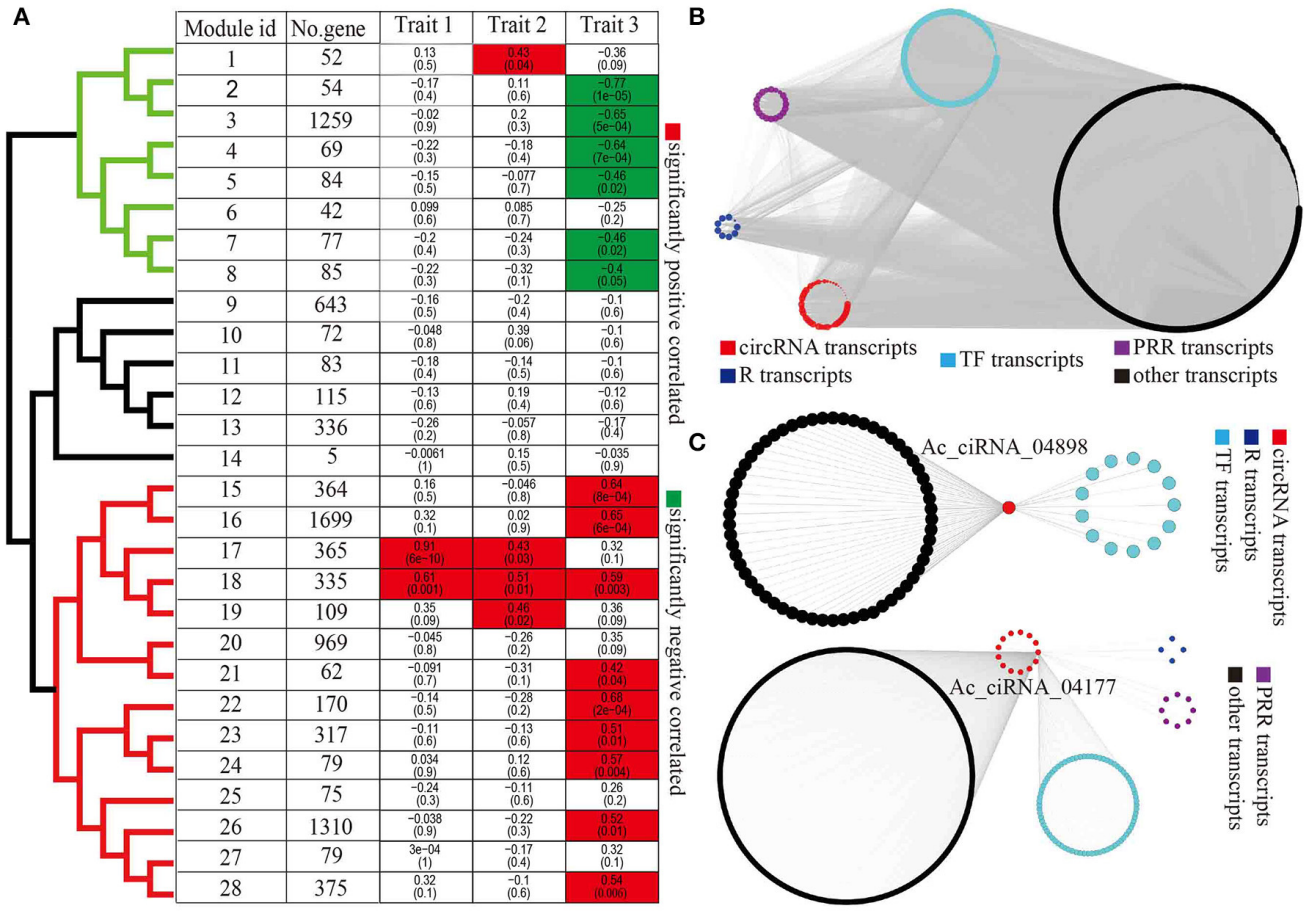
**WGCNA of circRNAs and protein-coding genes. (A)** Correlation between module eigengenes and biological traits. Modules were clustered based on eigengenes. The upper numbers were the correlations of modules and traits and lower numbers were *p*-values inside boxes. **(B)** An example of module network visualization (module 16). Blue, purple, cyan, black, and red nodes represent R genes, PRR genes, TFs, other genes, and circRNAs, respectively. **(C)** Two of examples of subnetwork of circRNAs (Ac_ciRNA_04898 and Ac_ciRNA_04177) and correlated genes. Blue, purple, cyan, black, and red nodes represent the R genes, PRR genes, TFs, other genes, and circRNAs, respectively.

To infer the potential functions of circRNAs, particularly circRNAs with possible roles in response to Psa infections, we performed WGCNA to systematically identify gene sets associated with a specific biological feature or process. We first combined the FPKM matrix of protein-coding transcripts and the RPM matrix of circRNA transcripts that were differentially expressed in at least one pairwise comparison from the taxon-dataset, yielding 8,700 protein-coding transcripts and 584 circRNA transcripts. After filtering transcripts with missing values, we identified a total of 28 modules (gene co-expression networks) with an average of 331.6 transcripts per module (Figure 4A). We clustered modules into three clades (clades in black, green, and red color, Figure 4A) on the basis of module eigengenes. Further we calculated the correlations between module and each of the three traits (traits 1, 2, and 3, Supplementary Table 8), resulting in significant positive or negative relationships that were observed 18 modules (Figure 4A). Amongst these, interestingly, modules 17 and 18 were simultaneously significantly associated with multiple traits, thereby suggesting that circRNAs also play a role in a wide variety of traits (Figure 4A). In addition, 15 out of 28 modules were correlated to species (trait 3), thereby indicating that kiwifruit circRNAs elicited species-specific responses to Psa invasion (Figure 4A). Furthermore, modules negatively and positively correlated to species were grouped into two distinct clades (green and red clades, Figure 4A).

With a particular interest in diversity in species resistance/susceptibility to Psa pathogens, we visualized the co-expression networks of species-associated modules (trait 3) using Cytoscape, which generated gene clusters that included the R genes, PRR genes, and TFs that were directly related to plant defense responses, as well as the co-expressed circRNAs (Figure 4B). The observed linear relationships between circRNAs and other diverse gene clusters were suggestive of complex molecular interactions underlying common biological and defense functions (Figure 4B, Supplementary Figure 4). GO enrichment analysis for protein-coding genes within species-specific modules indicated that Psa infections induced species-specific alterations in the expression profiles of kiwifruit genes that were related to photosynthesis, signal transduction, and immune responses (Supplementary Table 9). Furthermore, transcripts within modules that were negatively and positively correlated to species were involved in photosynthesis and immune responses, respectively (Supplementary Table 9). For instance, genes within module 8 that were negatively correlated to species were involved in the processes of photosynthesis and chlorophyll biosynthesis (Supplementary Table 9). In addition, the gene set in module 16 that was positively correlated to species were strongly enriched in respiratory burst, which is involved in defense response, including innate immune response, systemic acquired resistance, salicylic acid biosynthetic, jasmonic acid-mediated signaling pathway, response to chitin, regulation of plant-type hypersensitivity response, innate immune response, and negative regulation of programmed cell death (Supplementary Figure 5A).

On the basis of the co-expression relationship between protein-coding genes and circRNAs, we inferred the potential functions of circRNAs in kiwifruit plants in relation to Psa infections. Using the results of WGCNA, we constructed a subnetwork that had a specific circRNA at its focus or center. We retrieved gene sets (including protein-coding genes and circRNA genes) that were directly connected to the same circRNAs. Within each subnetwork, we retained protein-coding genes (hereby referred to as circRNA-associated protein-coding genes, CAPC). Several CAPC genes were R and PRR genes or TFs, which were directly involved in plant defense response, thereby suggesting that the related circRNA in the same subnetwork plays a similar function (Figure 4C). We performed GO enrichment analysis for all identified CAPC genes, which further indicated widespread enrichment of genes in relation to plant-pathogen interactions (Supplementary Table 10), particularly for circRNAs within species-specific modules. These results suggested that circRNAs regulated kiwifruit resistances/susceptibility to Psa in a species-specific manner. The circRNAs Ac_ciRNA_04898 and Ac_ciRNA_04177 were included in modules 18 and 16, respectively. Module 18 was significantly correlated to all the three traits, and module 16 was significantly correlated to species, which indicated that the two modules were closely related to kiwifruit response to Psa invasion. The CAPCs of both Ac_ciRNA_04898 and Ac_ciRNA_04177 were enriched in respiratory burst, which is involved in defense response, MAP kinase activity, and intracellular signal transduction (Figure 4C and Supplementary Figure 5), thereby suggesting the important roles of circRNAs in regulating kiwifruit responses to Psa infection. To validate the expression patterns of circRNAs and correlation between circRNAs and corresponding CAPCs, we conducted qPCR experiments for six circRNAs (Ac_ciRNA_04898/Ac_ciRNA_04177/Ac_ciRNA_13367/Ac_ciRNA_01028/Ac_ciRNA_11237/Ac_ciRNA_01629) and corresponding CAPCs (Achn034691/Achn058261/Achn026311/Achn144381/Achn084221/Achn060191). Our results indicated that qPCR results were highly correlated to RNA-seq results (average correlation was 0.99404, Supplementary Figure 6) and circRNAs were highly correlated to corresponding parent-genes (average correlation was 0.8908, Supplementary Figure 6).

## Discussion

Recent genomic studies have revealed widespread and diverse circRNAs in both animals and plants with potential regulatory function. However, reports on the characteristics and genome-wide distribution of circRNAs in non-model plants are limited (Memczak et al., 2013; Fan et al., 2015; Lu et al., 2015; Ye et al., 2015). Therefore, the molecular mechanisms and functions underlying the circRNAs in plants remain largely unknown. Additionally, functional and feature analysis of circRNAs illustrate that circRNAs can affect transcription in animals (Hansen et al., 2013). Conservation and expression analysis indicate that circRNAs are closely associated with plant development and stress response (Lu et al., 2015; Ye et al., 2015). In the present study, we showed the widespread expression of circRNAs in kiwifruit tissues, particularly that of the intergenic-circRNAs. Mechanisms including AS events are likely prevalent for the production of kiwifruit circRNAs, which are highly tissue- and taxa-specific. We found that AS events involving kiwifruit leaves were relatively stable during Psa invasion (Figure 1C), and most AS events occurred in protein coding-originating circRNAs (Supplementary Figure 2). These results indicate that kiwifruit circRNAs can regulate the expression of kiwifruit protein-coding genes that are of indispensable function. Moreover, we found that circRNAs can be significantly associated with *Actinidia* taxa with divergent responses to Psa infection, as well as reflect complex regulatory networks in which circRNAs are a critical player in host-pathogen interactions. Our results provide new insights into circRNAs in non-model plants, in particular the potential role of circRNAs that are associated with plant defense responses to pathogen invasion.

Compared to circRNAs identified in both *O. sativa* and *A. thaliana*, the total number of circRNAs in kiwifruit is relatively lower (3,582 vs. 6,012 and 12,037, respectively), which may be attributable to our more restrictive filter conditions (Ye et al., 2015). After circRNA identification using CIRI, we manually filtered 4,582 circRNAs, which were observed at only one biological replicate. In *O. sativa* and *A. thaliana*, exonic circRNAs showed the highest proportion (50.5, 85.7, respectively), yet in kiwifruit, intergenic circRNAs had the highest proportion (64.01%; Ye et al., 2015), thereby suggesting variations in circRNAs among different species. Consistent with the expression patterns in animals, *O. sativa*, and *A. thaliana*, our identified circRNAs in kiwifruit showed significant tissue-/stress-specific expression patterns (Figure 2), thereby illustrating the diverse roles of plant circRNAs in various biological processes. The present study identified several (59.67%) circRNAs that were only expressed in one tissue, a feature that has been reported in human and mouse (Gao et al., 2015; Venø et al., 2015). Interestingly, circRNAs in three *Actinidia* taxa revealed species-specific expression patterns during Psa infection (Figure 2). A total of 584 circRNAs were differentially expressed during Psa invasion (Supplementary Table 5). These results at least indicated that circRNAs participated in plant resistance/susceptibility to pathogen invasion.

In *O. sativa*, the expression profiles of 349 exonic circRNAs were significantly and positively correlated to that of their parent genes (Ye et al., 2015), and the expression of circRNAs were positively or negatively correlated to that of their parent genes with a *p* < 0.05 (Supplementary Table 6) in our case, thereby indicating that circRNA can up- and downregulate the expression of their parent genes. The decreased effects of circRNAs on their parent genes may be caused by emulative AS events during circRNA biogenesis (Chen, 2016). However, both exonic circRNA and intronic circRNA tended to be positively correlated to their parent genes (Figure 3B) thereby suggesting that most circRNAs were “enhancers” of their parental genes. Previous studies have verified that both exonic and intronic circRNAs can increase the expression of their parent genes in animals via *cis*-acting elements (Hansen et al., 2013; Li et al., 2015). More interestingly we found that intronic circRNAs had more remarkable regulatory effects on their parent genes comparing to that of exonic circRNAs (Figure 3). This difference may be determined by its own exonic and intronic properties. Exons are translated into proteins, and the biogenesis of exonic circRNAs may affect translation via emulative AS events. On the other hand, introns are spliced out during the post-transcriptional processing of mRNA and are thus not translated. Thus, employing products that originate from introns to regulate the expression of protein-coding genes is a more economical and efficient approach. CircRNAs can increase or decrease the expression levels of corresponding parent genes and this regulatory effect requires further investigation and verification.

Functional studies on circRNAs are limited, particularly in plants. Here, we employed WGCNA to investigate the co-expression network of circRNAs and protein-coding genes and further inferred the potential functions of circRNAs (Langfelder and Horvath, 2008). Consistent with the result of sample clustering for taxon-dataset (Figure 2), 15 out of 28 modules were significantly correlated to trait 3, thereby reflecting species-specific relationships (Figure 4A). In particular, we had identified a set of circRNAs that were observed within species-specific modules and showed direct associations with protein-coding genes that are involved in plant immune response, including multiple circRNAs that were directly connected to plant defense-related R and PRR genes (Figure 4 and Supplementary Figure 4). All of these findings collectively revealed the possible role of circRNAs in regulating host-pathogen interactions. To our limited knowledge, this could represent the first report on the correlation between circRNAs and plant resistance/susceptibility to bacterial pathogen invasion.

In summary, the present study has determined that circRNAs are co-opted for diverse distinct biological processes (Supplementary Table 10), thereby suggesting that circRNAs are extremely complicated regulators, including the crosstalk between different circRNAs that are co-expressed and commonly involved in the transcriptional regulatory network in eukaryotes. Further functional characterization of circRNAs in diverse taxa, as well as variable biological processes, are therefore needed to fully elucidate the role of circRNAs in plants.

## Author contributions

HH and YL directed the study. ZW and YL designed the experiments. LL and DL contributed to sample and tissue collection. ZW and SW performed the RNA-seq and cicrRNA identification pipeline. ZW, YL, QZ, and SW performed the data processing. ZW performed the quantitative RT-PCR experiments. ZW, YL, and HH drafted the manuscript. All authors approved the final draft.

## Funding

This research was partially supported by the National Natural Science Foundation of China (grant nos. 31471847 and 31572092), the Science and Technology Service Network Initiative of Chinese Academy (grant no. KFJ-EW-STS-076) and the Crop Germplasm Conservation Project Foundation of China (grant no. 2016NWB027). The funders had no role in study design, data collection and analysis, decision to publish, or preparation of the manuscript.

### Conflict of interest statement

The authors declare that the research was conducted in the absence of any commercial or financial relationships that could be construed as a potential conflict of interest.
