# Supplementary material for: Identification of Circular RNAs in Kiwifruit and Their Species-Specific Response to Bacterial Canker Pathogen Invasion
Source: Front Plant Sci. 2017 Mar 27;8:413. doi: 10.3389/fpls.2017.00413 (PMC5366334; doi:10.3389/fpls.2017.00413)
Supplement: Supplementary file 1 [file Image1.PDF]

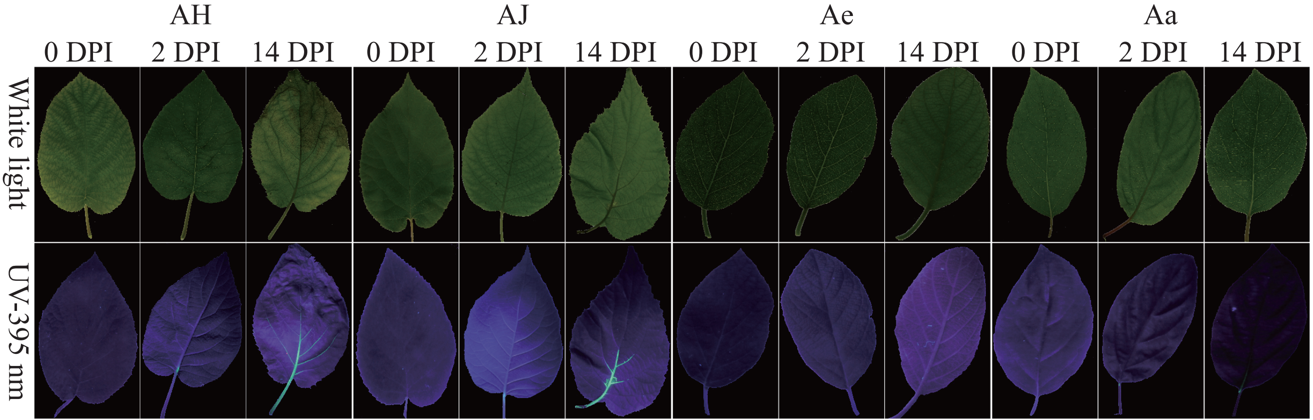

Supplementary Figure S1 Leaf symptoms of kiwifruit infected with GFPuv-labeled Psa strain C48. Leaf symptoms of kiwifruit samples during Psa infection. Photographs were taken under natural light (upper panels) and UV light (395 nm, low panels).
