# Supplementary material for: Identification of Circular RNAs in Kiwifruit and Their Species-Specific Response to Bacterial Canker Pathogen Invasion
Source: Front Plant Sci. 2017 Mar 27;8:413. doi: 10.3389/fpls.2017.00413 (PMC5366334; doi:10.3389/fpls.2017.00413)
Supplement: Supplementary file 2 [file Image2.PDF]

(A)

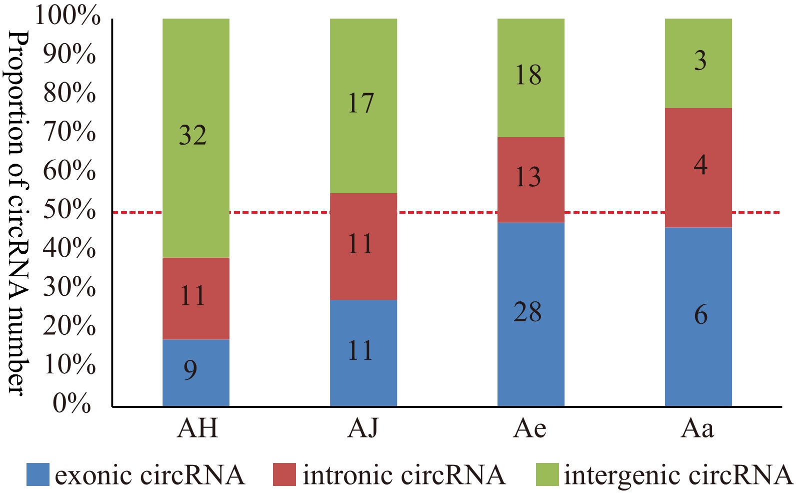

(B)

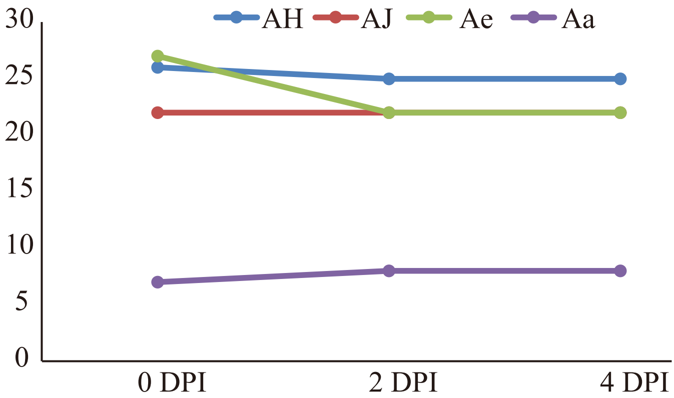

Supplementary Figure S2 The distribution of AS events among different types of circRNAs and at various sampling stages. (A) The distribution of AS events among different circRNA types. The blue, red and green bars represent exonic, intronic and intergenic circRNAs respectively. (B) The distribution of AS events among various sampling stages of different materials. The blue, red, green and purple lines represent AH, AJ, Ae and Aa respectively.
