# Supplementary figures and images for: Identification of Circular RNAs in Kiwifruit and Their Species-Specific Response to Bacterial Canker Pathogen Invasion
Source: Front Plant Sci. 2017 Mar 27;8:413. doi: 10.3389/fpls.2017.00413 (PMC5366334; doi:10.3389/fpls.2017.00413)

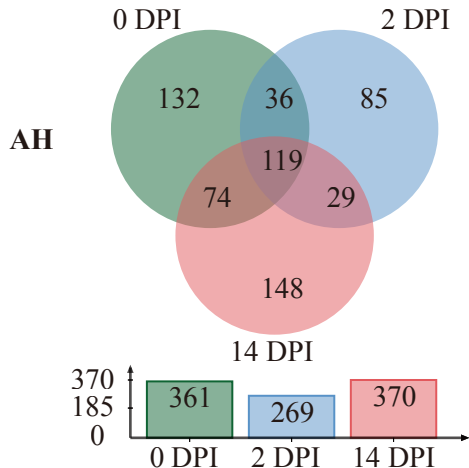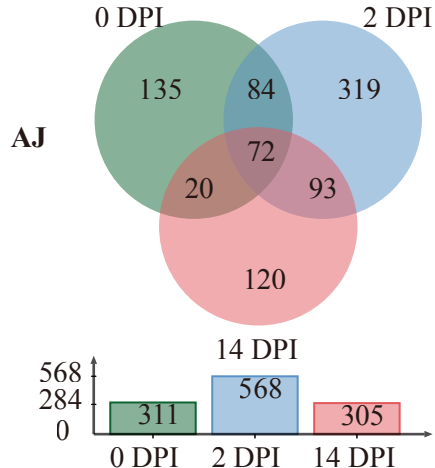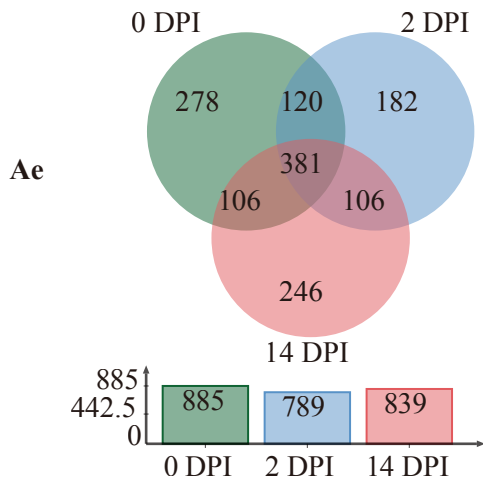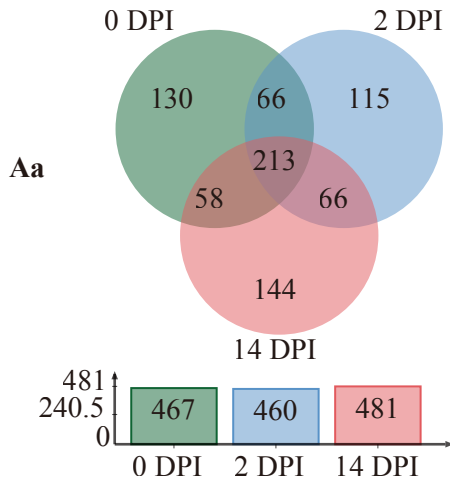

**Supplementary Figure S3** Venn diagram of circRNA distribution at three stages of each material.

Supplement: Supplementary file 3 [file Image3.PDF]
