# Supplementary material for: Identification of Circular RNAs in Kiwifruit and Their Species-Specific Response to Bacterial Canker Pathogen Invasion
Source: Front Plant Sci. 2017 Mar 27;8:413. doi: 10.3389/fpls.2017.00413 (PMC5366334; doi:10.3389/fpls.2017.00413)
Supplement: Supplementary file 4 [file Image4.PDF]

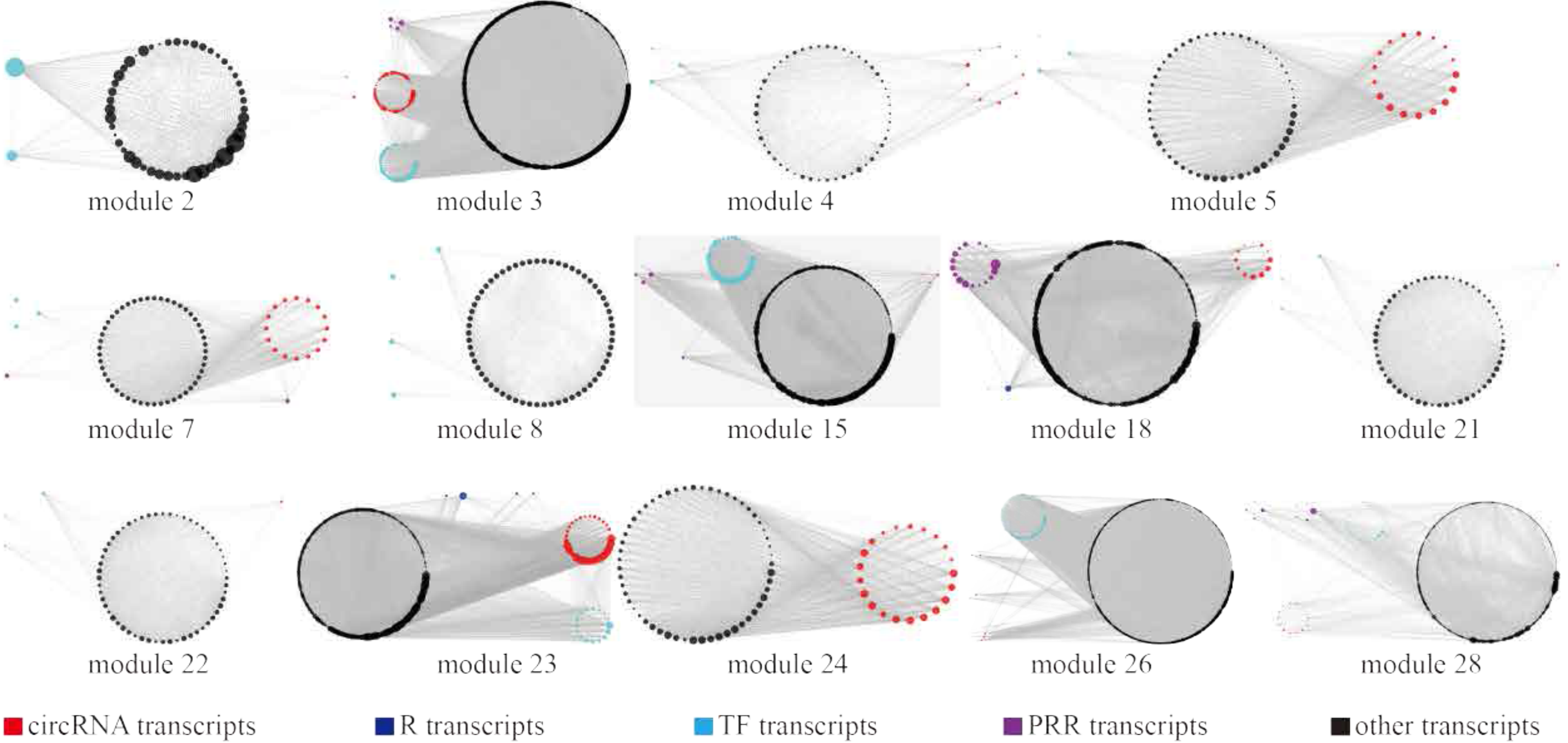

Supplementary Figure S4 Co-expression networks of species-specific modules identified. Blue, purple, cyan, black, and red nodes represent the R genes, PRR genes, TFs, other genes, and circRNAs, respectively.
