# Supplementary material for: Identification of Circular RNAs in Kiwifruit and Their Species-Specific Response to Bacterial Canker Pathogen Invasion
Source: Front Plant Sci. 2017 Mar 27;8:413. doi: 10.3389/fpls.2017.00413 (PMC5366334; doi:10.3389/fpls.2017.00413)
Supplement: Supplementary file 5 [file Image5.PDF]

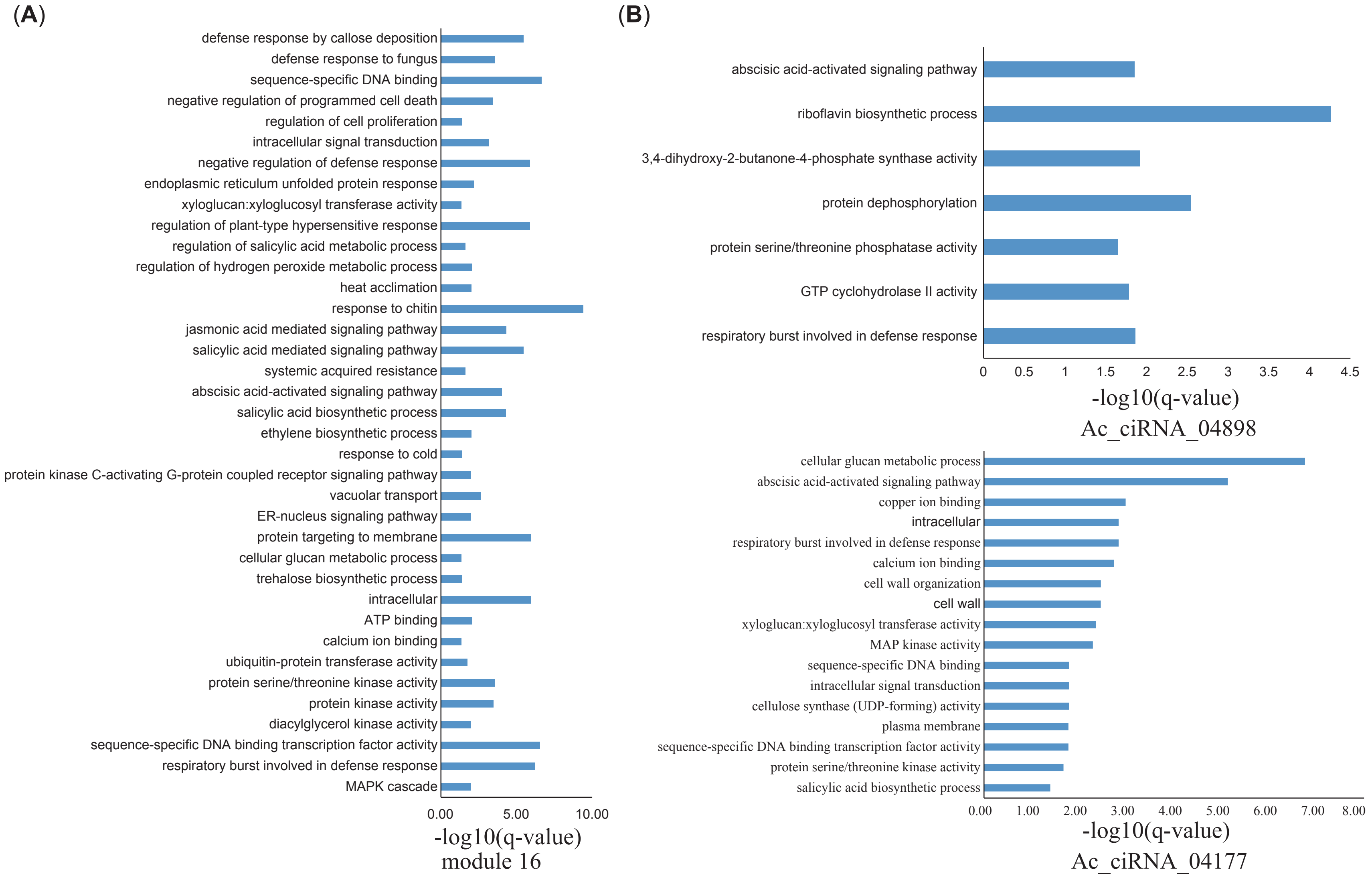


**Supplementary Figure S5** Examples of GO enrichment for co-expression network. (A) GO enrichment for the gene set within module 16. (B) GO enrichment for gene sets associated with Ac_ciRNA_04898 and Ac_ciRNA_04177.
