# Supplementary material for: Identification of Circular RNAs in Kiwifruit and Their Species-Specific Response to Bacterial Canker Pathogen Invasion
Source: Front Plant Sci. 2017 Mar 27;8:413. doi: 10.3389/fpls.2017.00413 (PMC5366334; doi:10.3389/fpls.2017.00413)
Supplement: Supplementary file 6 [file Image6.PDF]

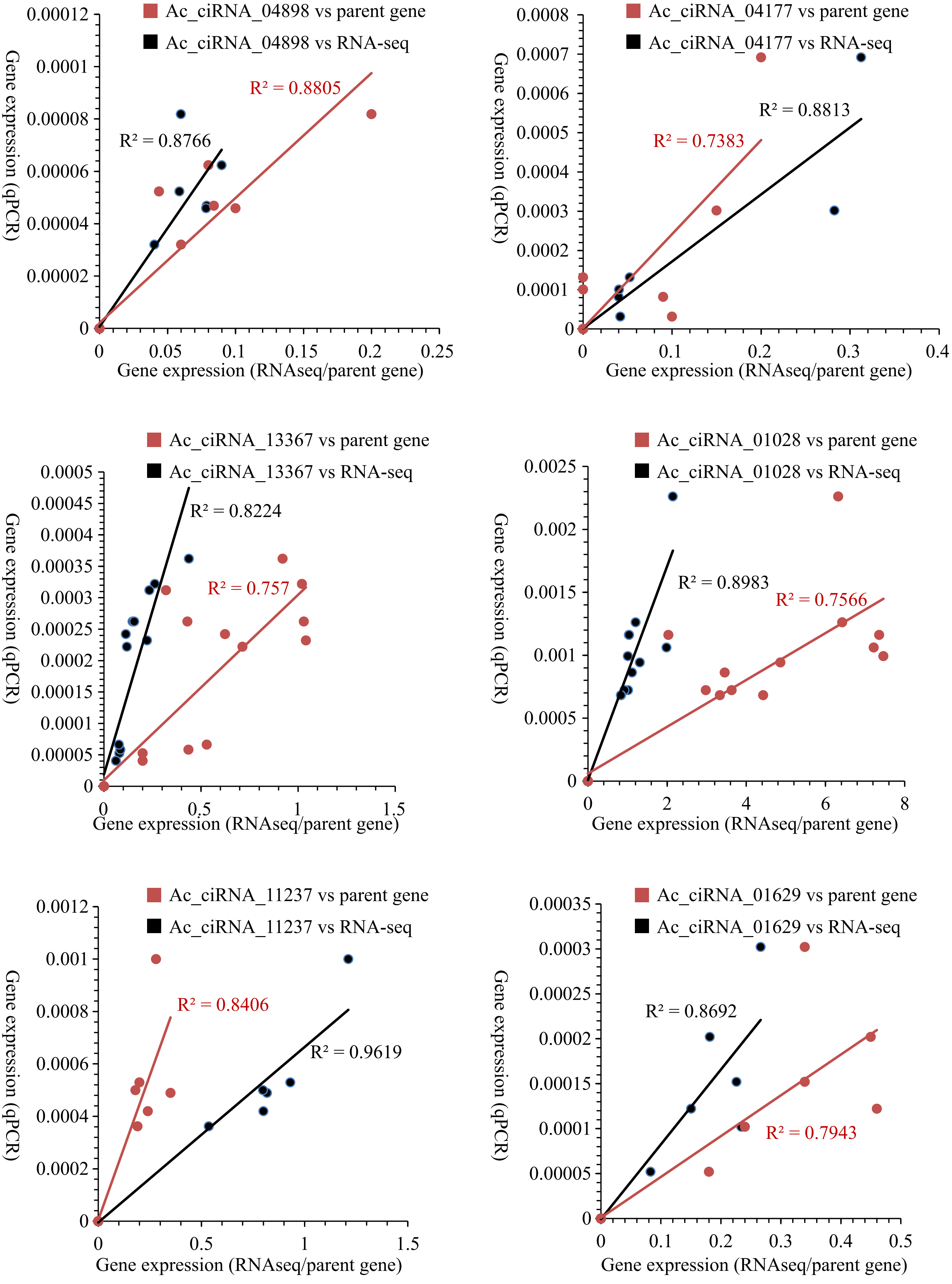

Supplementary Figure S6 circRNAs expression validation using qPCR. The black and red trendline represent the correlations of qPCR results and RNA-seq results of circRNA, qPCR results of circRNA and parent gene respectively.
